# IGF-1R down regulates the sensitivity of hepatocellular carcinoma to sorafenib through the PI3K / akt and RAS / raf / ERK signaling pathways

**DOI:** 10.1186/s12885-023-10561-7

**Published:** 2023-01-25

**Authors:** Wenpeng Cai, Yongfang Ma, Li Song, Niandie Cao, Jiafeng Gao, Shuping Zhou, Xiaolong Tang

**Affiliations:** 1 Medical school, Anhui University of Science and Technology, 232001 Huainan, China; 2 Medical School, Anhui University of Science & Technology, Class 8, Grade 18, Clinical Major, 232001 Huainan, China; 3 Institute of Environment-friendly Materials and Occupational Health of Anhu University of Science and Technology (Wuhu), 241003 Wuhu, China

**Keywords:** Insulin-like growth factor-1, Insulin-like growth factor-1 receptor, Sorafenib, Hepatocellular carcinoma, PI3K / akt, RAS / raf / ERK, Podophyllotoxin

## Abstract

**Background:**

Insulin-like growth factor-1 receptor (IGF-1R) promotes cell proliferation and migration and inhibitsapoptosis, all of which can contribute to the development of cancers.

**Method:**

This study investigated the effect and mechanism of IGF-1R in mediating the desensitization of hepatocellular carcinoma (HCC) to sorafenib.

**Results:**

IGF-1R, highly expressed in the HCC cell lines SK-Hep1 and HepG2, promotes cell proliferation, migration, and anti-apoptosis through PI3K / Akt and RAS / Raf / ERK signaling pathways, resulting in HCC resistance to sorafenib. Knockdown of IGF-1R by RNA interference decreased proliferation and cell migration and upregulation of sorafenib-induced apoptosis of HCC cells. In vivo studies demonstrated that IGF-1R knockdown inhibited the growth of SK-Hep1 xenografts.

**Conclusion:**

These data are evidence that IGF-1R participates in regulating the survival and cell growth of HCC through the PI3K / Akt and RAS / Raf / ERK signaling pathways. Intervention in the expression of IGF-1R may increase the inhibitory effect of sorafenib on HCC.

**Supplementary information:**

The online version contains supplementary material available at 10.1186/s12885-023-10561-7.

## Introduction

Hepatocellular carcinoma (HCC), the main subtype of primary liver tumors, causes the fourth largest number of cancer-associated deaths every year, which poses a great threat to human safety and health [1, 2]. Due to the rapid development of HCC, most tumors have progressed to advanced stage of liver cancer when they are diagnosed. Systemic therapy, such as with targeted drugs, is the first choice for the treatment of advanced HCC, mainly to reduce patients’ pain and prolong survival [3].

Sorafenib, a multi-target tyrosine kinase inhibitor, had anti-angiogenic and anti-proliferative effects in patients with advanced HCC, and it prolonged the median survival. Sorafenib inhibits the proliferation of tumor cells by inhibiting the Raf / MEK / ERK signaling pathway. It also inhibits vascular endothelial growth factor receptor, platelet-derived growth factor receptor β, and hepatocyte factor receptor (c-kit) to inhibit tumor angiogenesis. Thus, sorafenib has become a first-line drug for the treatment of advanced HCC [4, 5]. Although sorafenib has good efficacy in the initial stage of treatment, only about 30% of patients can benefit from sorafenib because patients usually acquire drug resistance within 6 months [6]. To enhance the antitumor effect of sorafenib, it is urgent to clarify its potential mechanism and determine the therapeutic target.

Insulin-like growth factor-1 receptor (IGF-1R) plays an important role in the regulation of cell differentiation and proliferation by activating downstream signaling pathways [7, 8]. IGF-1R overexpression is associated with an increased risk of developing cancers, including HCC [9, 10], and the expression and activation of IGF-1R are related to drug resistance in tumors. For example, IGF/IGF-1R signaling pathway directly mediated cell proliferation and survival [11], and IGF-1R is often overexpressed in liver cancer tissues [12]. IGF/IGF-1R mediates cell survival and induces tumor cell invasion and metastasis by crosstalk with the STAT3 signaling pathway, thereby enhancing tumor cell invasion and metastasis, and IGF/IGF-1R can induce cell migration through the PKD or RhoA/PKC signaling pathway [13]. Therefore, IGF-1R and molecules involved in the IGF/IGF-1R signaling pathway are reasonable targets for HCC treatment.

In this study, IGF-1R highly expressed in HCC cells conferred HCC resistance to sorafenib by activating the PI3K / Akt / and RAS / Raf / ERK signaling pathways. Downregulation of IGF-1R expression or inhibition of IGF-1R activation reversed the resistance of HCC to sorafenib and inhibited the growth of xenografts in vivo. Therefore, the combination of sorafenib and inhibition of IGF-1R activation may be beneficial for the treatment of HCC and provide a new therapeutic approach.

## Materials and methods

### Cell lines and reagents

Normal human hepatocytes THLE-2 and HHL-5 and human HCC cell lines Huh7, HepG2, and SK-Hep1 were purchased from the American Type Culture Collection (Manassas, VA, USA). The cells were cultured in RPMI-1640 (Hyclone; Salt Lake City UT, USA) and 10% fetal bovine serum (FBS) and maintained at 37 °C with 5% CO_2_. IGF-1 was purchased from Peprotech (Shanghai, China), sorafenib (#HY-10201S2) from Medchem Express (Monmouth Junction, NJ, USA), and podophyllotoxin (PPP) from Sigma Aldrich (St. Louis, Missouri, USA). The solvent/vehicle for PPP, IGF-1, and Sorafenib was ultrapure water, and the samples were stored at -20 °C. The lentivirus vector was from Sangonbiotech Genechem (Shanghai, China). IGF-1R, phospho-IGF-1R(p-IGF-1R), Akt, phospho-Akt (Ser473) β-actin, mTOR, phospho- mTOR (Ser2481), MEK1/2, phospho-MEK1/2, ERK1/2, phospho-ERK1/2, cleaved-caspase3, caspase3, poly ADP-ribose polymerase (PARP), cleaved PARP, horseradish peroxidase-coupled goat anti-rabbit and anti-mouse antibodies were purchased from Cell Signal Technology (Danvers, MA, USA). EdU and CCK-8 were purchased from Sijiqing Biological Engineering Materials Co., Ltd. (Hangzhou, China). JC-1 was obtained from Sigma-Aldrich (St. Louis, MO, USA). Hoechst 33,258 and Alexa fluor® 488 phalloidin were purchased from Labgic Technology Co., Ltd. (Biosharp, Hefei, China).

### Cell scratch healing experiment

Cells in exponential growth period were collected after trypsin digestion and diluted to 2.5 × 10^5^ cells/mL. Six-well plates, marked on the back and containing 2 mL of cell suspension per well, were cultured at 37℃ and 5% CO_2_. After 24 h, the plates were scratched with a 10µL pipet tip held perpendicular to a horizontal line on the bottom surface. The plates were washed with PBS buffer 3 times to remove cell debris. Two milliliters of serum-free medium and corresponding drugs for treatment were added for various times. Wound size ratio % (24 or 48 h) = Wound size (0 h) - Wound size (24 or 48 h) / Wound size (0 h). Photographs were taken under an optical microscope, and cell mobility was calculated.

### Cell clone formation experiment

Cells in logarithmic growth stage were digested with trypsin and blown into single cells. Cell suspensions were inoculated in six-well plates at a density of 1000 cells per well and placed in a cell incubator for 10 days. Medium was removed from the plates, and the plates were washed with PBS 3 times. The cells were fixed in 4% paraformaldehyde for 20 min, washed with PBS 3 times, dyed with 0.1% crystal violet dye for 10 min, photographed, and counted.

### EdU cell proliferation experiment

Cells at 2 × 10^5^ cells per well were cultured in 24-well plates overnight. Cells in each well were treated with corresponding drugs for 24 h and incubated for 2 h after 10 µL of EdU working solution were added to each well. The culture medium was removed, and the cells were fixed with 4% paraformaldehyde for 15 min. The cells were washed with PBS 3 times, incubated with Triton X-100 at room temperature for 15 min, and washed with PBS 3 times. Click reaction solution was added to cover the sample evenly and incubated at room temperature for 30 min. Photographs were taken under an inverted fluorescence microscope, and the cell proliferation rate was calculated.

### Lentivirus transfection

1.5 × 10^5^ cells per well were added to 24-well plates and cultured overnight. The culture medium was removed, and a mixture of 250 µL culture medium and lentivirus, with an MOI value of 30, was added to the wells. Incubation was continued for 4 h, and 250 µL culture medium was added. The next day, the medium containing virus was removed, fresh culture medium was added, and incubation was continued.

### Transwell test

Cell migration was measured with Transwell chamber (JET BIOFIL, Guangzhou, China). 3 × 10^5^ HCC cells, resuspended in 100 µL RPMI 1640, were inoculated into the upper chamber; 600 µL RPMI 1640, supplemented with 10% FBS, were added into the lower chamber. After 24 h of culture, the cells in the upper layer were wiped off with a wet cotton swab. The cells on the other side of the membrane were stained with crystal violet, photographed, and counted under a microscope.

### Acridine orange/ethidium bromide staining

Acridine orange (AO) working solution, 100 µg/mL, (Solarbio, Beijing, China) and ethidium bromide (EB) (Keygene Biotech, Nanjing, China) were mixed in the ratio of 1:1, diluted 20 times with PBS, mixed evenly, made into working solution, and added dropwise to the cell climbing tablet. The morphology of apoptotic cells was analyzed and counted by inverted fluorescence microscope. The percentage of apoptotic cells = (total number of apoptotic cells / total number of cells counted) × 100%。.

### Indirect immunofluorescence

After the cells had adhered to the wall overnight, they were fixed with methanol for 30 min and washed with PBS. 200 µL IGF-1R specific antibody were added and incubated overnight at 4℃. The slides were washed with PBS, and Alexa fluor ® 488 phalloidin, 200 µL, were added and incubated in the dark for 30 min, and the slides were washed with PBS 3 times. Hoechst 33,258 was added and reacted for 10 min in the dark. The slides were washed with PBS and photographed under an inverted fluorescence microscope.

### Immunocytochemistry

After incubation for 24 h, the cells were fixed with methanol and washed with PBS. Peroxidase blocker was added dropwise and incubated at 37℃ for 15 min. The slides were washed with PBS 3 times, and nonimmune animal serum for blocking of nonimmune proteins was added at 37℃ for 15 min. The serum was discarded, and 200 µL of IGF-1R specific antibody was added dropwise and incubated overnight at 4℃. After PBS wash, 200 µL of secondary antibody was added and incubated at 37℃ for 15 min; after PBS washing 3 times, 100 µL of horseradish-labeled streptomycin working solution was added. After incubation at 37℃ for 15 min and PBS wash 3 times, freshly prepared diaminobenzidine chromogenic solution was added, and the slides were counterstained with hematoxylin, rinsed with running water, and photographed under the upright fluorescence microscope.

### Mitochondrial membrane potential detection

1.5 × 10^5^ cells were placed in each well in the 24-well plates and cultured overnight. After drug treatment, 200 µL of JC-1 working solution was added to each well and incubated for 20 min. After PBS wash 3 times, Hoechst 33,342 staining was added and reacted away from light for 10 min. After cleaning with PBS, photographs were taken under inverted fluorescence microscope. In normal mitochondria, JC-1 aggregates in the mitochondrial matrix to form a polymer, which fluoresces strongly red; in unhealthy mitochondria, due to the decrease or loss of membrane potential, JC-1 exists only as a monomer in the cytoplasm, which fluoresces green.

### Western blotting

Cells were treated with various drugs, and cells were collected to extract proteins. Separation gel was prepared and concentrated, and protein was loaded for electrophoresis. The separation gel was removed, and the membrane (Millipore, USA) was transferred for protein assay. The membrane was blocked with 5% skim milk for 1 h, then placed in the primary antibody solution at 4 ℃ overnight. The membrane was washed with TBST for 3 times. The bands were incubated with HRP-conjugated secondary antibody for 30 min, and the gray value was analyzed by Image J version 1.48 software.

### Cell counting kit-8 experiment

1.5 × 10^3^ cells per well were inoculated in 96-well plates overnight. After 24 h of drug treatment, 10 µL CCK-8 solution was added to each well and incubated in the cell incubator for 2 h. The absorbance was measured at 450 nm, and the cell viability was calculated by Graphpad Prism version 5.0 software. (Graphpad, La Jolla, CA, USA).

### Animal experiments

The animal experiments were approved by the ethics committee of Medical College of Anhui University of technology. 100 µL SK-Hep1, SK-Hep1 cells transfected with siRNA2-IGF-1R lentivirus (SK-Hep1^siRNA2 − IGF−1R^), and SK-Hep1 cells transfected with siRNACtrl-IGF-1R lentivirus (SK-Hep1^siCtrl − IGF−1R^) suspension (2 × 10^7^ cells/ml) were injected subcutaneously into the right dorsum of 5-week-old female BALB/c nude mice (Vital River Laboratories, Beijing, China). The mice injected with SK-Hep1 cell suspension were randomly divided into 3 groups: (1) control group, 100 µL normal saline per day; (2) sorafenib group, sorafenib 30 mg/kg per day; (3) PPP + sorafenib group, PPP 10 mg/kg + sorafenib 30 mg/kg per day. SK-Hep1^siRNA2 − IGF−1R^ and SK-Hep1^siCtrl − IGF−1R^ tumor-bearing mice were fed 30 mg/kg sorafenib daily. Each group contained five mice. The solvent for PPP, IGF-1, and sorafenib was saline, and Ctrl groups were injected with the same amount of saline. Each group of mice received treatment for 3 weeks, and the tumor size and body weight were measured every 3 days. Tumor volume was calculated according to the formula V = L×W^2^ × 1/2 (V, volume; L, tumor length; W, tumor width).

### Statistical analysis

SPSS 18.0 software (SPSS Inc., Chicago, IL, USA) and Graphpad Prism 5.0 software were used for data analysis. All experiments were repeated at least 3 times, and the results were expressed as mean ± standard deviation (SD). One-way ANOVA was used to test the differences between groups. *p* < 0.05 was statistically significant.

## Results

### IGF-1R is highly expressed in HCC cells

**Fig. 1:**
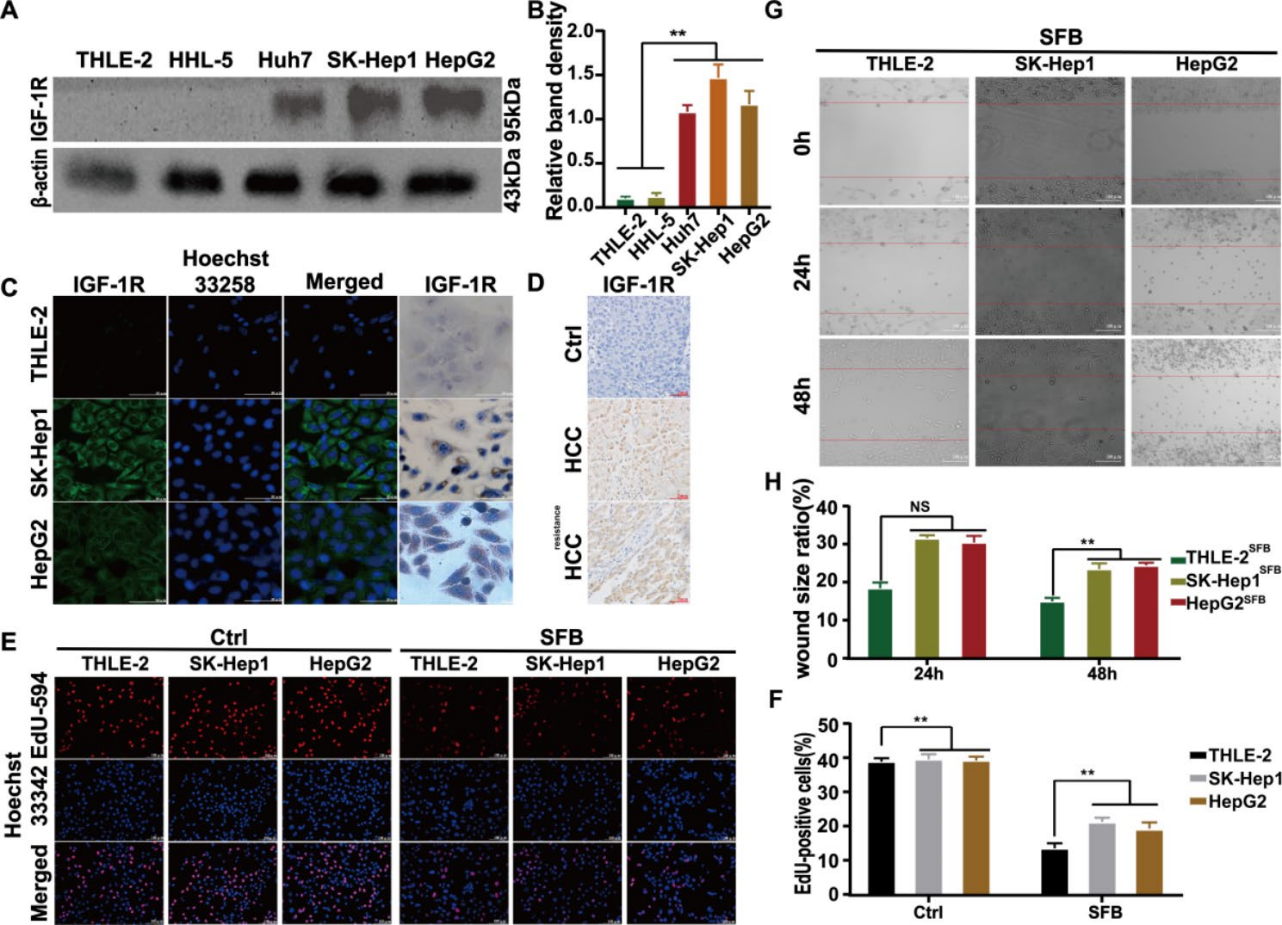
IGF-1R is highly expressed in HCC cells. (**A**, **B**) Western blot detected the expression level of IGF-1R in each cell line. (**C**) The expression of IGF-1R in SK-Hep1 and HepG2 cell lines was detected by indirect immunofluorescence and immunocytochemistry assays (×200). (**D**) The expression of IGF-1R in tissues was detected by immunocytochemistry assay (×200). (**E**, **F**) The difference in proliferation level between THLE-2 and HCC cells was determined with EdU proliferation experiments (magnification: 200×). (**G**, **H**) The scratch healing rate and magnification of THLE-2 and HCC cells at 0, 24, and 48 h after scratch were recorded under an inverted microscope (× 200)

Western blot experiments showed that the expression level of IGF-1R was lower in normal hepatocyte lines THLE-2 and HHL-5 than in HCC cell lines SK-Hep1, HepG2, and Huh-7, where it was highest in SK-Hep1 (Fig. 1 A and 1B). The results of indirect immunofluorescence and immunocytochemistry experiments confirmed that HCC cells highly expressed IGF-1R. IGF-1R was mainly located in cell membranes and cytoplasm, as shown in Fig. 1 C. In vivo, IGF-1R expression was significantly higher in HCC tissues than in HCC adjacent tissues (Fig. 1D). We compared the higher and lower expression of IGF-1R, as shown in Fig. 1E F. EdU experiments showed that the proliferation ability of SK-Hep1 and HepG2 cells with high expression of IGF-1R was significantly higher than in THLE-2 cells with low expression of IGF-1R after treatment with sorafenib (^**^p < 0.01). The results of the cell scratch test showed that the scratch healing rate of SK-Hep1 and HepG2 cells at 24 and 48 h was also significantly higher than that of THLE-2 cells after treatment with sorafenib (^*^p < 0.05) (Fig. 1G H). Therefore, the proliferation and migration abilities of Huh7, SK-Hep1, and HepG2 cell lines were significantly higher than those of THLE-2 and HHL-5 cell lines, which may be due to the higher expression of IGF-1R in HCC cells.

### Downregulation of IGF-1R expression restored the inhibitory effect of sorafenib on the proliferation of HCC cells

**Fig. 2:**
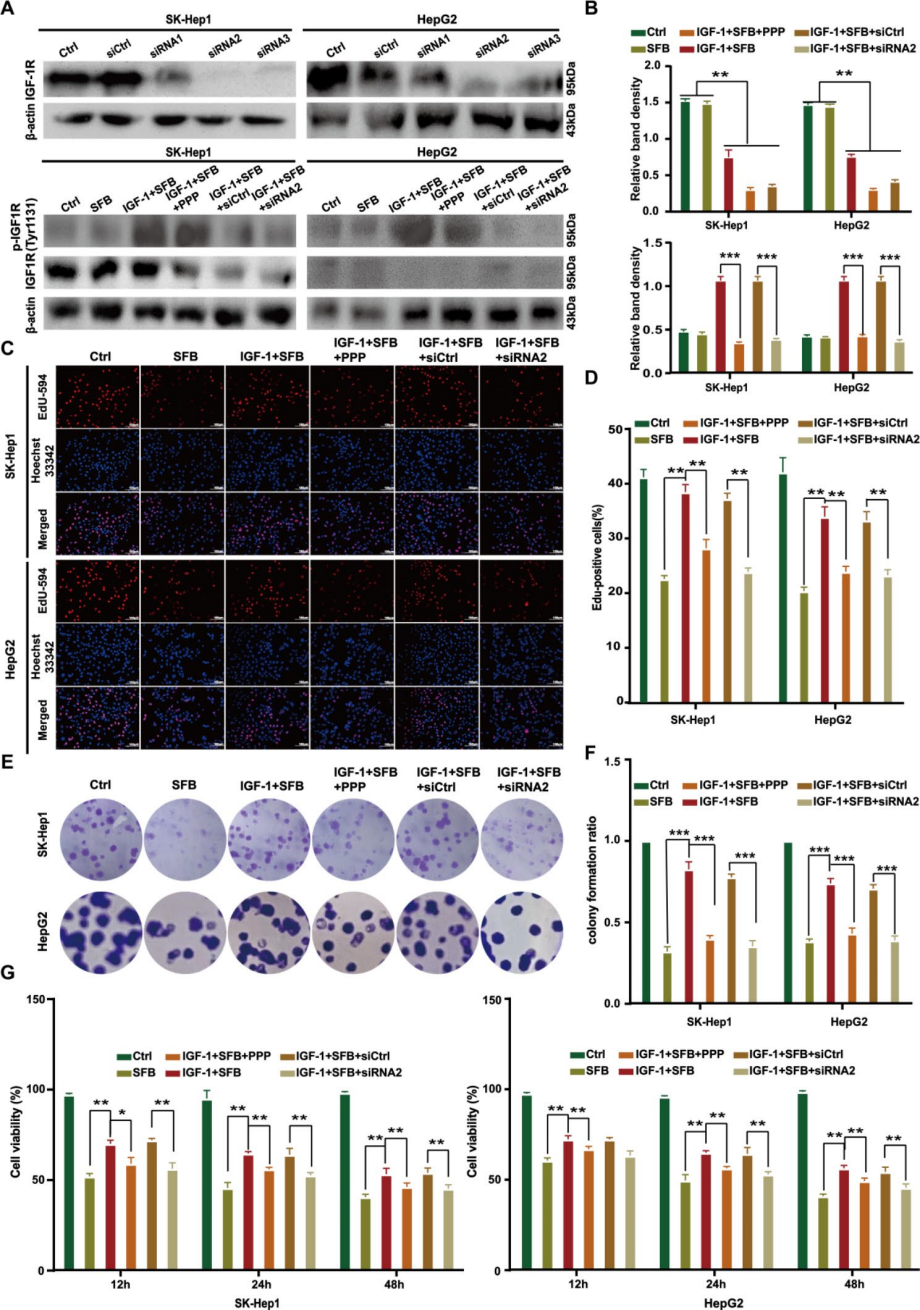
Down regulation of IGF-1R expression and activation restored the inhibitory effect of sorafenib on the proliferation of HCC cells. (**A**, **B**) Western blot detected the expression level of IGF-1R and p-IGF1R in HCC cells. (**C**, **D**) HCC cells were exposed to each drug treatment and stained with EdU (×200). The cell proliferation rate was calculated as the percentage of EdU-positive nuclei in the total nuclei. (**E**, **F**) The cell clones in the six-well plate were stained with 0.1% crystal violet dye. The clones were counted, and representative photos of the clones were taken. (**G**) HCC cells were exposed to each drug treatment. The absorbance was measured, and the cell viability was calculated. Sorafenib (4 µM), IGF-1 (100 ng/mL), and PPP (100 nM). The statistics chart represents the mean ± SD determined in triplicate

To investigate the correlation between IGF-1R and cell proliferation, we transfected HCC cells with siRNA IGF-1R lentivirus to knock down the expression of IGF-1R. Seventy-two hours after transfection, the cellular proteins of each group were extracted, and the siRNA IGF-1R lentivirus with the best efficiency of interfering with IGF-1R was screened by Western blot. Results are shown in Fig. 2 A and Fig. 2B, where siRNA2 IGF-1R lentivirus interfered with IGF-1R expression with the highest efficiency (^**^p < 0.01). Therefore, HCC cells stably transfected with siRNA2 IGF-1R lentivirus, were selected for subsequent experiments. The effect of IGF-1R on the anti-proliferation effect of sorafenib on HCC was analyzed with EdU and cell clone experiments **(**Fig. 2 C, 2D**)**. The results showed that the proliferation rate of SK-Hep1 cells in the single-drug sorafenib (4 µM) treatment chamber was significantly lower than in the blank control group (^**^p < 0.01), indicating that sorafenib inhibited the proliferation of HCC cells; compared with single-drug sorafenib (4 µM) treatment, the cell proliferation rate of IGF-1 (100 ng/mL) + sorafenib (4 µM) was increased (*p* < 0.01); however, when HCC cells were treated with IGF-1 (100 ng/mL) + sorafenib (4 µM) + IGF-1R inhibitor (PPP, 100 nM) or IGF-1 (100 ng/mL) + sorafenib (4 µM) + siRNA2 (IGF-1R), the proliferation of HCC cells was decreased significantly compared with that of IGF-1 (100 ng/mL) + sorafenib (4 µM); the results demonstrated that IGF-1R is involved in the regulation of cell proliferation and reverses the inhibition of proliferation of HCC cells by sorafenib. The cell clone formation experiment yielded similar results **(**Fig. 2E F**)**: IGF-1 (100 ng/mL) downregulated the inhibition of sorafenib (4 µM) on the clone formation efficiency of HCC cells, whereas the inhibition of sorafenib on the clone formation of HCC cells was restored to varying degrees with treatment of IGF-1 (100 ng/mL) + sorafenib (4 µM) + IGF-1R inhibitor (PPP, 100 nM) or IGF-1 (100 ng/mL) + sorafenib (4 µM) + siRNA2 (IGF-1R) (^***^p < 0.001). The CCK-8 results also showed that the cell viability of HCC cells was decreased in a time-dependent manner after sorafenib treatment for 12, 24, and 48 h, and IGF-1 (100 ng/mL) decreased the cytotoxicity of sorafenib (4 µM) to HCC cells. When IGF-1 (100 ng/mL) + sorafenib (4 µM) + IGF-1R inhibitor (PPP, 100 nM) or IGF-1 (100 ng/mL) + sorafenib (4 µM) + siRNA2 (IGF-1R) were treated together, the effect of IGF-1 on reducing the cytotoxicity of sorafenib cells was decreased to varying degrees (Fig. 2G). These results documented that downregulating the expression of IGF-1R and inhibiting the activation of IGF-1R could increase the ability of sorafenib to inhibit the proliferation of HCC cells.

### Downregulation of IGF-1R expression or inactivation inhibits cell migration

**Fig. 3:**
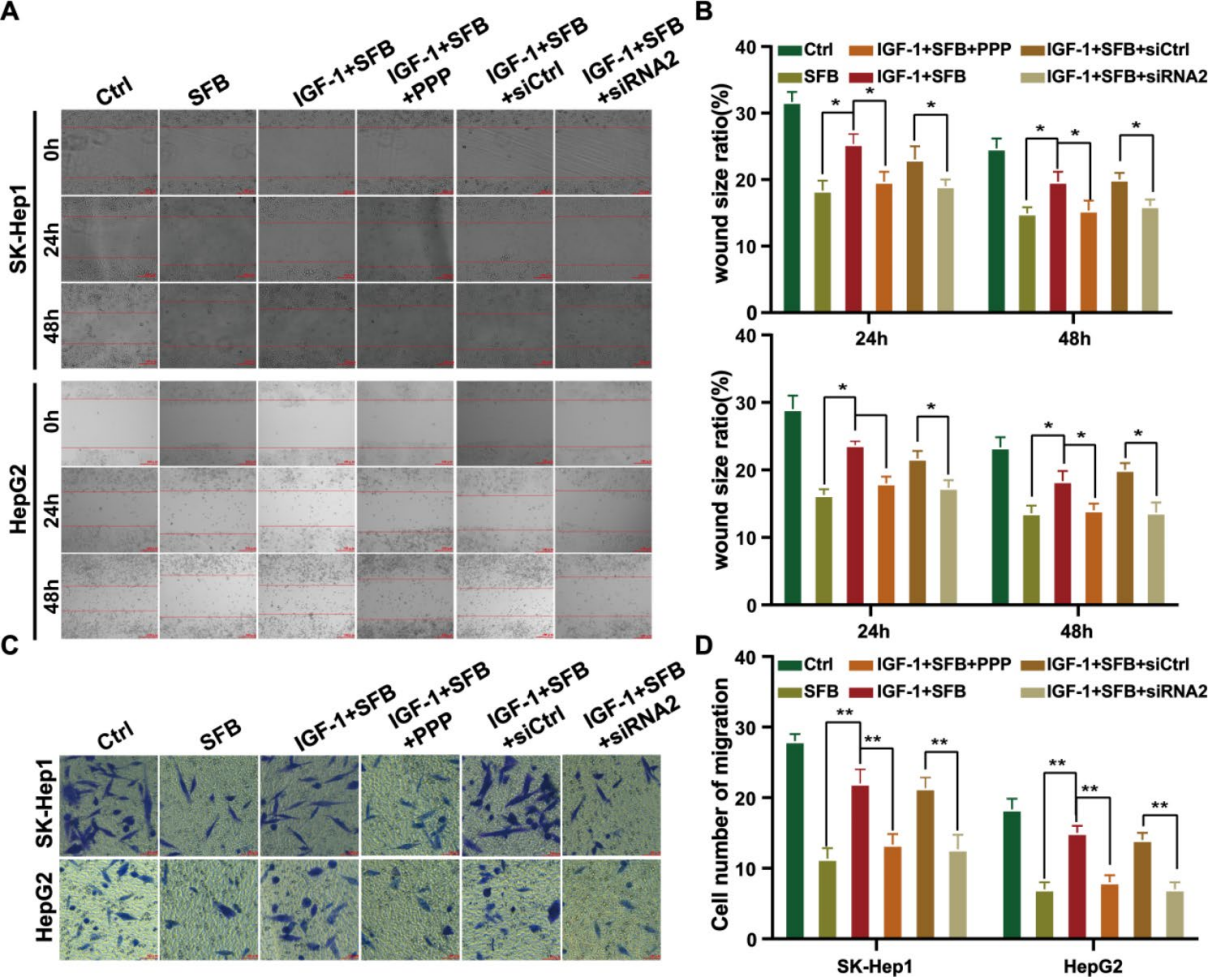
IGF-1R affects the migration of HCC cells. (**A**, **B**) Representative cell photomicrographs of scratch healing of HCC cells in each treatment group at 0, 24, and 48 h (×200). (**C**, **D**) The number of migrating cells passing through Transwell ventricular membrane in each treatment group

To investigate the effect of IGF-1R on HCC cell migration, SK-Hep1 and HepG2 cells were treated with single drug sorafenib: IGF-1 (100 ng/mL) + sorafenib (4 µM), IGF-1 (100 ng/mL) + sorafenib (4 µM) + PPP (100 nM) or IGF-1 (100 ng/mL) + sorafenib (4 µM) + siRNA2 (IGF-1R), and the cell scratch healing test and Transwell test were performed. The results showed that IGF-1 + sorafenib + PPP and IGF-1 + sorafenib + siRNA2 treatment inhibited the scratch healing rate (^*^p < 0.05) (Fig. 3 A, B). Transwell results also showed that IGF-1 + sorafenib + PPP and IGF-1 + sorafenib + siRNA2 treatment downregulated the migration of HCC cells compared with that of IGF-1 + sorafenib treatment (^*^p < 0.05) (Fig. 3 C, D). These results documented that PPP, an inhibitor of IGF-1R, or interference with the expression of IGF-1R inhibited the migration of HCC cells.

### Downregulation of IGF-1R expression or activation enhances the cytotoxicity of sorafenib on HCC

**Fig. 4:**
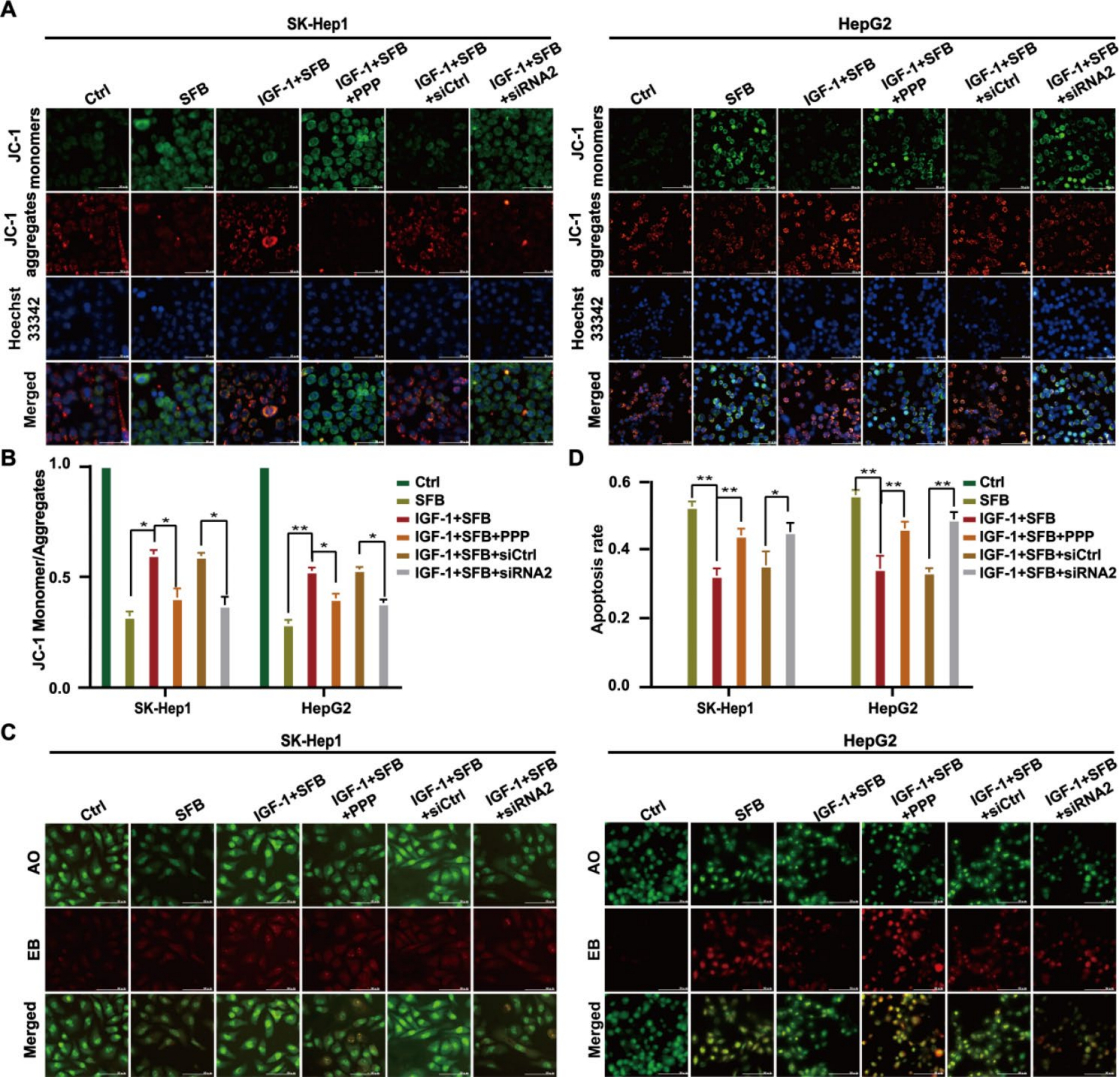
Downregulation of IGF-1R expression or inhibition of IGF-1R activation increases sorafenib toxicity to HCC cells. (**A**, **B**) JC-1 was analyzed to reflect the level of apoptosis in each group by detecting the changes of mitochondrial membrane potential (×200). (**C**, **D**) AO/EB staining was used to detect the level of apoptosis in each treatment group (×200)

To explore whether IGF-1R is involved in inducing the resistance of HCC cells to sorafenib, SK-Hep1 and HepG2 cells were treated with single-drug sorafenib (4 µM), IGF-1 (100 ng/ml) + sorafenib (4 µM), IGF-1 (100 ng/ml) + sorafenib (4 µM) + PPP (100 nM) and IGF-1 (100 ng/ml) + sorafenib (4 µM) + siRNA2 (IGF-1R) for 24 h, respectively. Compared with single-drug sorafenib, IGF-1 + sorafenib treatment reduced the decrease of mitochondrial potential induced by sorafenib in HCC cells; however, the mitochondrial potential of HCC cells was significantly decreased in treatments in which binding of PPP inhibited the activation of IGF-1R or binding of siRNA2 interfered with the expression of IGF-1R independent of IGF-1 (Fig. 4 A). AO/EB staining confirmed that combined PPP or siRNA2 treatment increased the apoptosis of SK-Hep1 cells (^*^p < 0.05). These results documented that downregulation of IGF-1R expression or inhibition of IGF-1R activation could increase the cytotoxicity of sorafenib to HCC.

### IGF-1R signaling mediates the resistance of HCC to sorafenib through the PI3K/Akt and Ras/Raf/ERK pathways

**Fig. 5:**
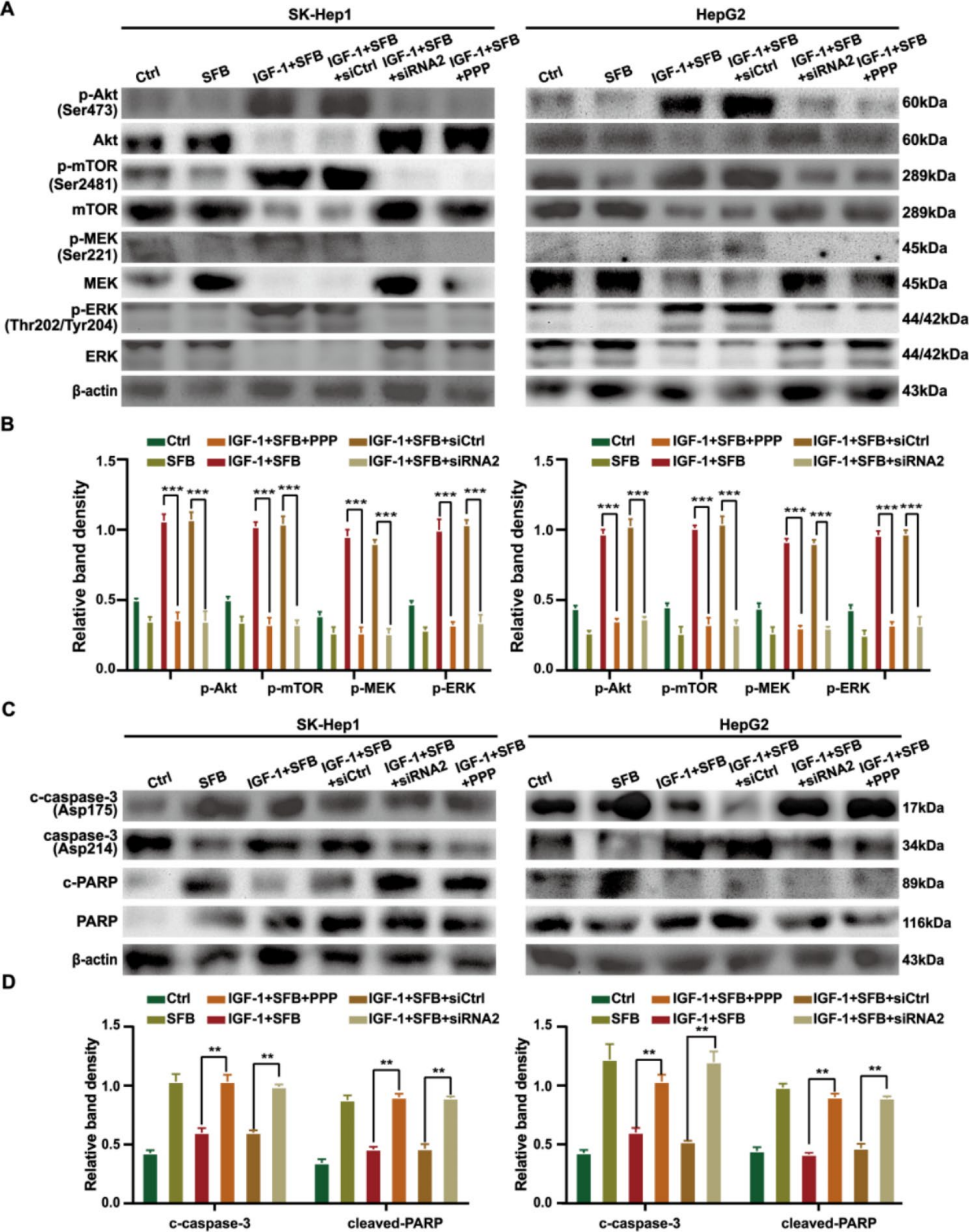
IGF-1R signaling regulates HCC resistance to sorafenib by activating PI3K/Akt and Ras/Raf/ERK pathways. (**A, B**) Changes of key kinases in IGF-1R/p-IGF-1R, PI3K/Akt and Ras/Raf/ERK pathways in each treatment group. (**C, D**) The levels of cleaved caspase 3 and cleaved PARP were detected by Western blot. The reference of each protein is β-actin

To explore the possible mechanism of IGF-1R-induced proliferation, migration, anti-apoptosis, and sorafenib resistance in HCC cells, we measured the phosphorylation level of related kinases in the main signal pathways of HCC cells by Western blot. The results showed that the level of p-IGF-1R increased after 24 h of IGF-1 treatment, and the phosphorylation levels of MEK, ERK, and Akt increased with the increase of IGF-1R phosphorylation (^**^p < 0.01). The results are illustrated in Fig. 5 A and 5B. When HCC cells were transfected with siRNA IGF-1R lentivirus, the expression levels of IGF-1R and p-IGF-1R decreased significantly, and the levels of p-Akt, p-mTOR, p-MEK and p-ERK also decreased, suggesting that the activation levels of PI3K/Akt and Ras/Raf/ERK signaling pathways were inhibited when IGF-1R expression was downregulated (^**^p < 0.01). In addition, compared with HCC cells treated with sorafenib and IGF-1 alone, p-IGF-1R was decreased significantly in HCC cells pre-treated with IGF-1R inhibitor PPP, and the activation levels of key kinases in PI3K/Akt and Ras/Raf/ERK signaling pathways were also significantly inhibited (^**^p < 0.01). Moreover, the levels of cleaved caspase 3 and cleaved PARP in sorafenib combined with siRNA IGF-1R group and PPP pretreatment group were significantly up-regulated; however, the level of apoptosis-related protein molecules in IGF-1 + sorafenib treatment group was significantly lower than that in the single drug sorafenib treatment group (^**^p < 0.01) **(**Fig. 5 C and 5D**)**, suggesting that IGF-1 stimulation downregulates the pro-apoptotic ability of sorafenib on HCC cells. Together, these results suggested that IGF-1R signaling leads to HCC resistance to sorafenib through activation of PI3K/Akt and Ras/Raf /ERK pathways.

### Downregulation of IGF-1R expression can restore the anti-hepatoma effect of sorafenib in vivo

**Fig. 6:**
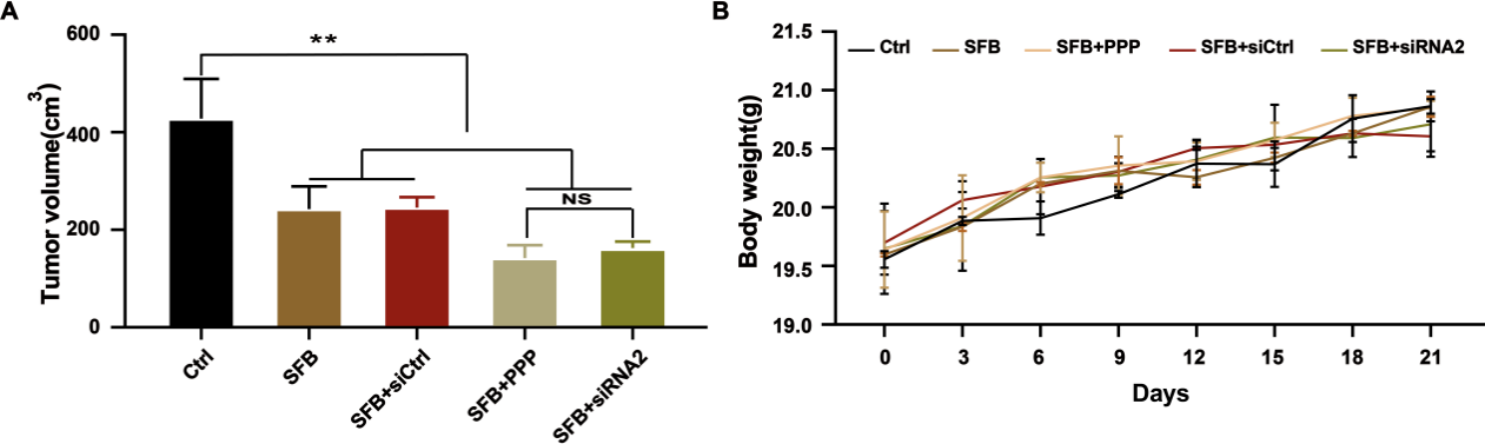
Downregulating the expression or activation of IGF-1R restored the antitumor effect of sorafenib in vivo. (**A**) The end-point tumor volume (mm^3^) of nude mice in each group was determined. (**B**) The body weight (g) of mice in each group was recorded every 3 days

After 21 days of drug treatment of xenograft-bearing mice, the grafts were isolated, the volume after drug treatment was measured, and the anti-tumor effect of each treatment was evaluated. The average volume of xenografts in the sorafenib group was significantly less than in control group. The average volume of xenografts in the siRNA2 + sorafenib group and the PPP + sorafenib group were significantly less than in the sorafenib group (^**^p < 0.01). The results are shown in Fig. 6 A. There was no significant difference in the body weight of mice in each group, indicating that the mice had good tolerance to the drugs, as shown in Fig. 6B. These results indicated that down regulating the expression and activation of IGF-1R enhanced the antitumor effect of sorafenib in vivo.

## Discussion

The tumors in most patients with advanced HCC that are treated with the first-line drug, sorafenib, develop drug resistance [14, 15]. Sorafenib-acquired resistance reportedly involves a variety of mechanisms, including multiple-signal pathway crosstalk; abnormal expression of tyrosine kinase receptors, such as PDGFR- β, c-kit, Flt-3, VEGFR and EGFR; epithelial-mesenchymal transition (EMT); and cancer stem-cell differentiation [16–18]. In this work, we demonstrated that IGF-1R decreased the sensitivity of HCC to sorafenib treatment; down-regulation of IGF-1R increased the inhibition of sorafenib on the migration of HCC cells mainly through PI3K/Akt and Ras/Raf/ERK pathways. The combination of inhibiting IGF-1R expression and administering sorafenib is synergistic in inhibiting the growth of HCC by regulating the PI3K / Akt and Ras / Raf / ERK pathways.

IGF-1R is a transmembrane tyrosine protein kinase receptor [18, 19]. When IGF-1R binds to the corresponding ligand, IGF-1, it catalyzes the phosphorylation of autophosphorylation sites and causes intracellular signal transduction [19, 20]. IGF-1R is highly expressed in a variety of tumors, including HCC, and is involved in biological effects such as tumor-cell proliferation, differentiation, invasion, metastasis, and anti-apoptosis [10, 20–22]. Our results are consistent with those that have been reported: Western blot confirmed that the expression level of IGF-1R in normal hepatocyte lines THLE-2and HHL-5 was very low, whereas its expression in HCC cell lines SK-Hep1, HepG2, and Huh-7 was higher. Compared with normal liver cell lines, HCC cell lines with high values of IGF-1R expression have significantly greater cell proliferation, migration, and anti-apoptosis, suggesting that the high expression of IGF-1R is important in the development of HCC and is related to the proliferation, migration, and anti-apoptosis of liver cancer cells.

To explore the mechanism of IGF-1R-mediated HCC-related biological functions, we designed three siRNAs targeting IGF-1R genes and transfected them into HCC cells by lentivirus. We found that lentivirus-mediated siRNA2 stably knocked down IGF-1R protein expression (Fig. 2 A). Therefore, we selected HCC cells with stably knockdown IGF-1R expression by siRNA2 as IGF-1R low-expression experimental cells. We used the cells to further explore the role of IGF-1R in downregulating sorafenib in the treatment of HCC in vitro and in vivo. In vitro functional studies revealed that siRNA downregulating IGF-1R expression and podophyllin inhibiting IGF-1R phosphorylation downregulated the proliferation and migration potential of HCC cells and enhanced sorafenib-induced apoptosis (Figs. 2, 3 and 4), which was consistent with published results [23–25].

Finally, by comparing the activation levels of major kinases involved in regulating cell proliferation, survival, and anti-apoptosis-related signal pathways in HCC cells, we explored the possible mechanisms of IGF-1R inducing HCC growth and invasion and IGF-1R downregulation of the response of HCC cells to sorafenib. We performed Western blot analysis to identify the kinases related to the function of IGF-1R. The results showed that the protein levels of p-Akt, p-mTOR, p-MEK, and p-ERK were decreased in IGF-1R knockdown HCC cells compared with levels in HCC cells (IGF-1R high expression). In addition, silencing IGF-1R combined with sorafenib synergistically enhanced the apoptosis of HCC cells through the caspase / PARP pathway, suggesting that IGF-1R is involved in the proliferation, migration, and anti-apoptosis of HCC cells through the PI3K / Akt and Ras / Raf / ERK signaling pathways and negatively regulating the inhibitory effect of sorafenib on HCC. IGF-1R is overexpressed in lung cancer, breast cancer, pancreatic cancer, colorectal cancer, and HCC [23–27]. It is associated with tumor prognosis, and crosstalk between IGF-1R and PI3K/Akt and Ras/Raf/ERK signaling pathways is an important factor in the progression and drug resistance of various tumors. However, as far as we know, this is the first systematic study of the association between IGF-1R and sorafenib in the treatment of HCC.

Although HCC resistance to SFB can be explained by the targeted activation of PI3K/Akt and RAS/RAF/ERK signaling pathways by IGF-1R, we cannot rule out the existence of other mechanisms, which need to be investigated in the future.

## Conclusion

This study showed that IGF-1R can decrease the response of hepatocellular carcinoma to sorafenib through PI3K / Akt and Ras / Raf / ERK signaling pathways. Silencing the expression of IGF-1R or inhibiting the activation of IGF-1R can inhibit the growth and invasion of HCC and enhance the response of HCC to sorafenib. Inhibiting the abnormal activation of IGF-1R and/or downregulating the expression of IGF-1R is a potential treatment strategy for HCC.

## Electronic supplementary material

Below is the link to the electronic supplementary material.

Supplementary Material 1: Western blotting original data

## Data Availability

The datasets supporting the conclusions of this article are included within its additional file.

## Abbreviations

HCC: hepatocellular carcinoma
PI3K: phosphatidylinositol 3-kinase
EMT: epithelial-mesenchymal transition
MEK: mitogen-activated protein kinase
ERK: extracellular signal-regulated kinase
IGF-1: Insulin-like growth factor-1
IGF-1R: Insulin-like growth factor-1 receptor
PBS: Phosphate-buffered saline
