# Supplementary material for: IGF-1R down regulates the sensitivity of hepatocellular carcinoma to sorafenib through the PI3K / akt and RAS / raf / ERK signaling pathways
Source: BMC Cancer. 2023 Jan 25;23:87. doi: 10.1186/s12885-023-10561-7 (PMC9875405; doi:10.1186/s12885-023-10561-7)

Figure 1A.
IGF-1R

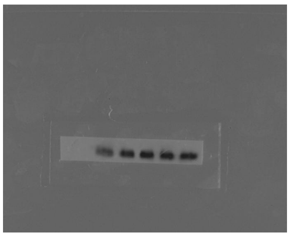

β-actin

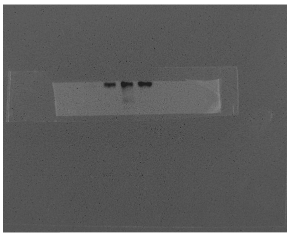

Figure 2A.
SK-Hep1 **HepG2**
β-actin

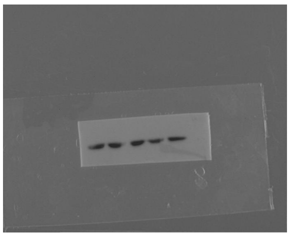

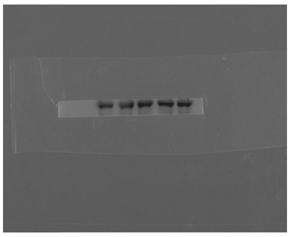

IGF-1R

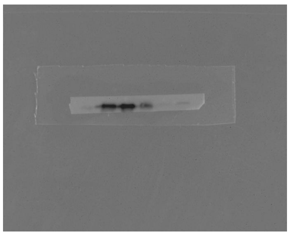

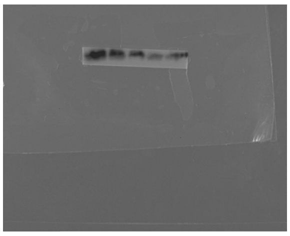

β-actin

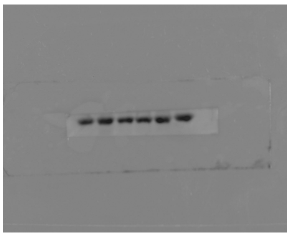

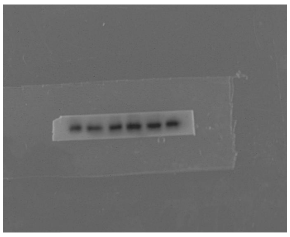

IGF-1R

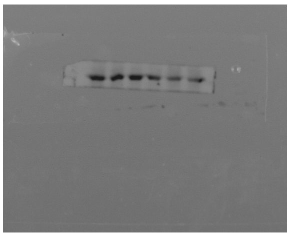
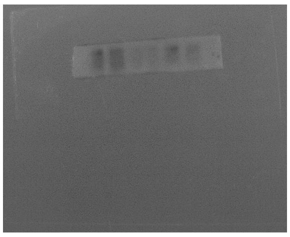

p-IGF-1R

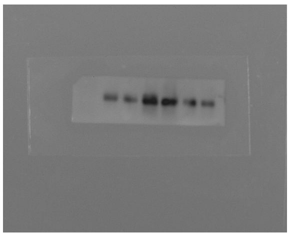
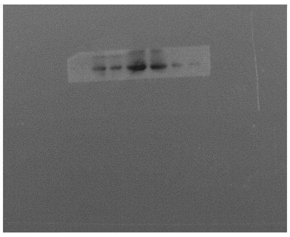

Figure 5A

SK-Hep1 HepG2

β-actin

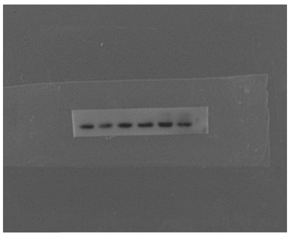
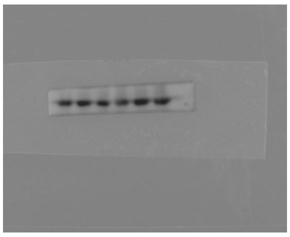

Akt

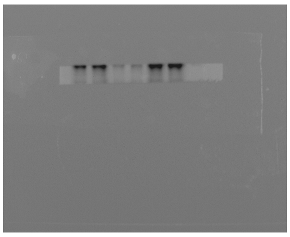
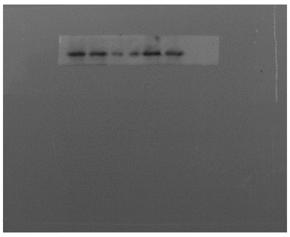

ERK

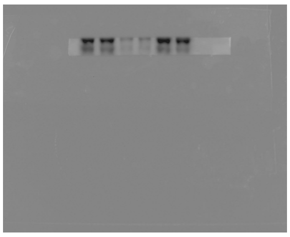
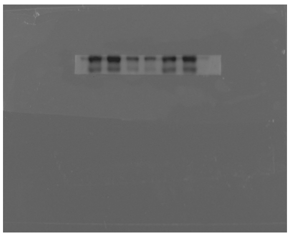

MEK

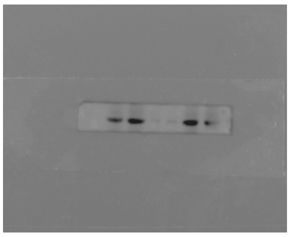
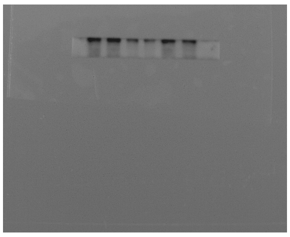

mTOR

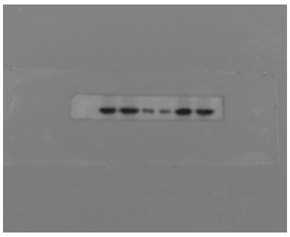
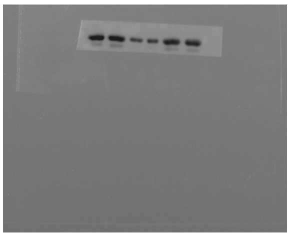

p-Akt

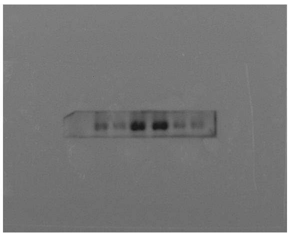
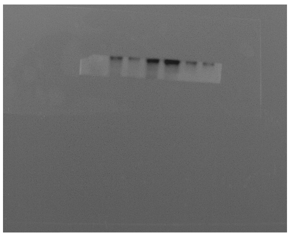

p-ERK

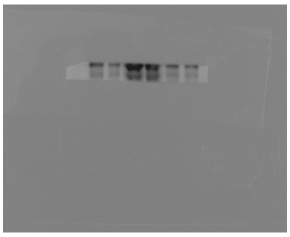
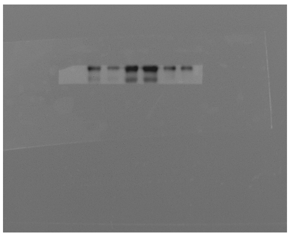

p-MEK

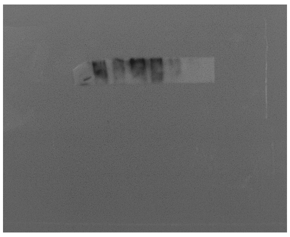
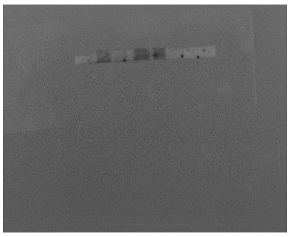

p-mTOR

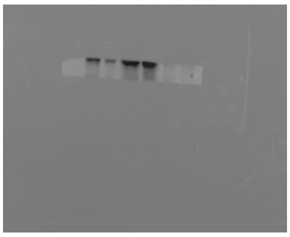
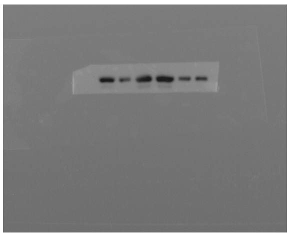

Figure 5C

β-actin

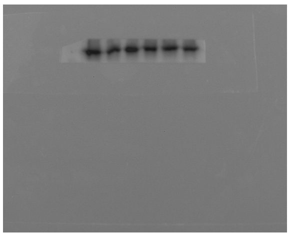
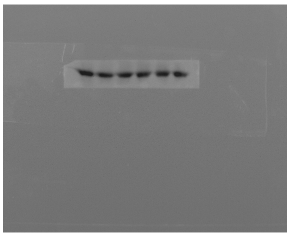

Caspase3

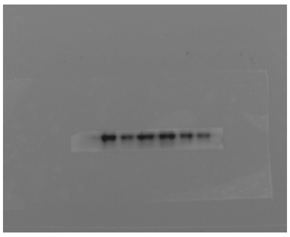
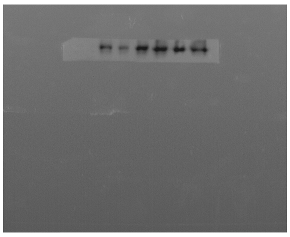

C-Caspase3

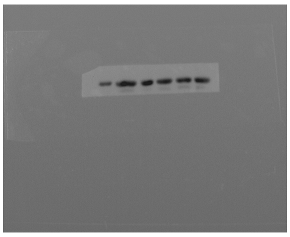
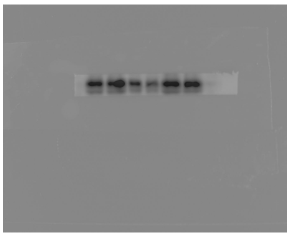

c-PARP

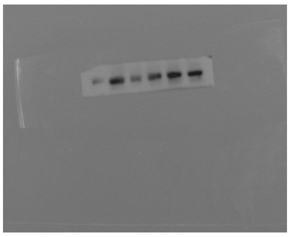
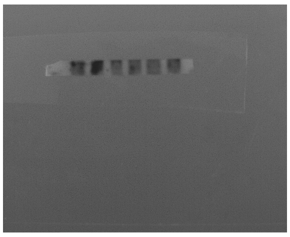

PARP

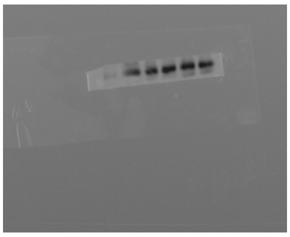
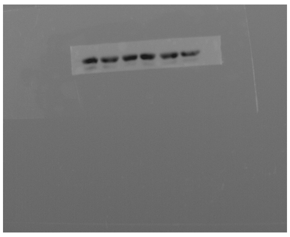

Supplement: Supplementary file 1 — Supplementary Material 1: Western blotting original data [file 12885_2023_10561_MOESM1_ESM.pdf]
